# *Pteridium spp*. and Bovine Papillomavirus: Partners in Cancer

**DOI:** 10.3389/fvets.2021.758720

**Published:** 2021-11-02

**Authors:** Beatriz Medeiros-Fonseca, Ana Lúcia Abreu-Silva, Rui Medeiros, Paula A. Oliveira, Rui M. Gil da Costa

**Affiliations:** ^1^Centre for Research and Technology of Agro-Environmental and Biological Sciences (CITAB), Inov4Agro, University of Trás-os-Montes and Alto Douro (UTAD), Vila Real, Portugal; ^2^Veterinary Sciences Department, University of Trás-os-Montes and Alto Douro (UTAD), Vila Real, Portugal; ^3^Veterinary Sciences Department, State University of Maranhão (UEMA), São Luís, Brazil; ^4^Molecular Oncology and Viral Pathology Group, Research Center of IPO Porto (CI-IPOP)/Rede de Investigação em Saúde (RISE)@CI-IPOP (Health Research Network), Portuguese Oncology Institute of Porto (IPO Porto)/Porto Comprehensive Cancer Center (Porto.CCC), Porto, Portugal; ^5^Molecular Oncology and Viral Pathology Group, Faculty of Medicine, University of Porto, Porto, Portugal; ^6^Biomedicine Research Center (CEBIMED), Faculty of Health Sciences, Fernando Pessoa University, Porto, Portugal; ^7^Virology Service, Portuguese Institute of Oncology (IPO-Porto), Porto, Portugal; ^8^LEPABE - Laboratory for Process Engineering, Environment, Biotechnology and Energy, Faculty of Engineering, University of Porto, Porto, Portugal; ^9^Post-graduate Programme in Adult Health (PPGSAD), Department of Morphology, Federal University of Maranhão (UFMA), UFMA University Hospital (HUUFMA), São Luís, Brazil

**Keywords:** papillomaviruses, bovine papillomavirus, cancer, molecular epidemiology ptaquiloside, bladder

## Abstract

Bovine papillomavirus (BPV) are a cause for global concern due to their wide distribution and the wide range of benign and malignant diseases they are able to induce. Those lesions include cutaneous and upper digestive papillomas, multiple histological types of urinary bladder cancers—most often associated with BPV1 and BPV2—and squamous cell carcinomas of the upper digestive system, associated with BPV4. Clinical, epidemiological and experimental evidence shows that exposure to bracken fern (*Pteridium* spp.) and other related ferns plays an important role in allowing viral persistence and promoting the malignant transformation of early viral lesions. This carcinogenic potential has been attributed to bracken illudane glycoside compounds with immune suppressive and mutagenic properties, such as ptaquiloside. This review addresses the role of BPV in tumorigenesis and its interactions with bracken illudane glycosides. Current data indicates that inactivation of cytotoxic T lymphocytes and natural killer cells by bracken fern illudanes plays a significant role in allowing viral persistence and lesion progression, while BPV drives unchecked cell proliferation and allows the accumulation of genetic damage caused by chemical mutagens. Despite limited progress in controlling bracken infestation in pasturelands, bracken toxins remain a threat to animal health. The number of recognized BPV types has steadily increased over the years and now reaches 24 genotypes with different pathogenic properties. It remains essential to widen the available knowledge concerning BPV and its synergistic interactions with bracken chemical carcinogens, in order to achieve satisfactory control of the livestock losses they induce worldwide.

## Introduction

Papillomaviruses (PVs) are small epitheliotropic viruses that contain circular double-stranded DNA with about 7,000 base pairs (bp) as genetic material and belong to the *Papillomaviridae* family (1, 2). PVs infect mammals, reptiles, birds and fish and can be found on healthy skin and mucous membranes, as well as in benign proliferative epithelial lesions and in invasive cancers (3–6). Structurally, PVs consist of a capsid with icosahedral symmetry composed of 72 capsomeres in forming star-shaped pentamers and measuring 55–66 nm. The viral forms a super helix with histoproteins derived from infected host cells (1, 2). The genome structure of all PVs has significant similarities. The circular genome is divided into three regions: one region containing genes that encode the initial viral proteins involved in viral replication and modulation of cellular functions called the early (“E”) region, the late (“L”) region which encodes the L1 structural protein (the main capsid protein) and the L2 minor capsid protein, and the long control region (LCR) region (1, 2, 5, 7). Difficulties in classifying PVs have been caused by their high genotypic diversity that goes far beyond those found in other virus families, and the high number of PV hosts. Recent phylogenetic studies of PVs demonstrate a variety of evolutionary processes that underlie this diversity (2, 8, 9). Currently, within the *Papillomaviridae* family, 53 genera are identified according to the International Committee on Taxonomy of Viruses (ICTV) (5). The inclusion of different species within each genus is done based on phylogenetic analysis, biological properties and similarity of *L1* gene nucleotide sequences, due to their conserved pattern within each taxonomic genus (4, 10). Thus, PVs that share at least 60% similarities in the *L1* gene are grouped into the same genus. A relationship has long been recognized between exposure to bovine papillomavirus (BPV) and certain toxic ferns—most commonly *Pteridium* spp.—leading to the development of malignant neoplasms, as previously reviewed (11, 12). The complex interplay between viral and chemical factors in the development of cancer in cattle is the subject of the present review.

## *Pteridium* spp. And Its Toxicity

**Figure 1:**
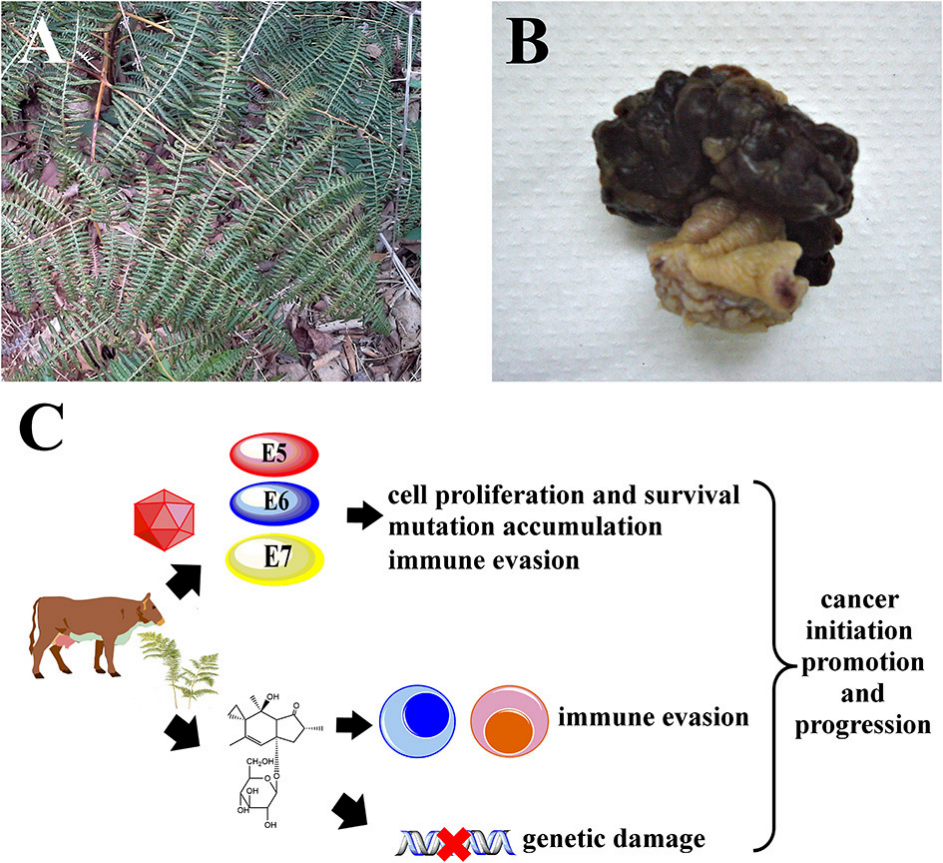
It is hypothesized that *Pteridium* spp. and BPV synergize to drive carcinogenesis in cattle. **(A)** Mature *Pteridium* sp. fronds, Maranhão, Brazil. **(B)** Representative macroscopic view of a bovine urinary bladder hemangiossarcoma. **(C)** Illudane glycosides like ptaquiloside promote immune evasion by inactivating critical leukocytes like cytotoxic T lymphocytes and NK cells, allowing viral persistence. Genetic damage caused by bracken toxins accumulates due to unchecked cell proliferation and survival induced by BPV E5, E6 and E7 oncoproteins, leading to cancer.

*Pteridium* spp. known as bracken fern or common fern (Figure 1A) is a pteridophyte belonging to the *Dennstaedtiaceae* family, and comprises multiple species and strains (13, 14). Bracken fern is distributed all over the world, including bush areas, forest undergrowth and cultivated land, but it preferentially develops in acidic and deep soils and in humid and shaded areas (15, 16). Bracken fern has a negative effect at the economic level since it directly impacts animal production. Such impacts can be classified in two ways: direct losses when there is death, reproductive disorders, weight loss or reduced growth and indirect losses which involve medical costs, habitat changes and alterations in the handling of animals (17). *Pteridium* spp. contains many bioactive substances such as quercetin (18), shikimic acid (19), tannins (20) and a group of toxic illudane glycosides among which ptaquiloside (21–23) has been best characterized, as previously reviewed by our group (11). Ptaquiloside was first identified in 1983 (21–23) as a water-soluble, unstable glycoside, which contains a reactive cyclopropane ring. Ptaquiloside reacts with water to form highly reactive intermediates which alkylate biomolecules such as DNA, triggering pathological cellular changes, as recently reviewed (24). Other related ferns such as *Cheilanthes sieberi* have been studied chemically and were also found to contain illudane glycosides (25). The concentrations of ptaquiloside and other illudane glycosides in bracken fern show wide seasonal variations and also depend on bracken strains, geographical location and the parts of the pant being analyzed (e.g., rhizomes vs. fronds), reaching a maximum of 18.81 mg/g of dry plant (26). Young sprouts, known as croziers, are the parts that contain more ptaquiloside (26–28). Illudane glycosides are responsible for the immunotoxic, mutagenic and carcinogenic properties of bracken fern (24). Other compounds like quercetin, initially thought to be involved in bracken fern-induced cancers (29, 30), were later shown to be safe *in vivo* (31, 32). The toxic effects of bracken fern vary according to the animal species, the dose ingested and the time of consumption (17). In grazing animals, bracken ingestion is associated with multiple syndromes. These include acute bracken poisoning and others that develop upon prolonged exposure, like thiamine deficiency in horses, progressive retinal degeneration of sheep, bovine enzootic hematuria and upper alimentary tract carcinomas, as previously reviewed (11). Early works from the 17th century already mention bracken's acute gastrointestinal toxicity when ingested (33). In ruminants, acute bracken poisoning can cause necrosis of the laryngeal, pharyngeal and intestinal mucosae, as well as severe leukopenia with neutropenia or lymphopenia, anemia and thrombocytopenia (34, 35). These effects have been experimentally reproduced in laboratory rats fed bracken and in cattle administered ptaquiloside (36, 37). Another fern, *Adiantopsis chlorophylla*, containing caudatoside, an illudane glycoside structurally related with ptaquiloside, has also been associated with acute poisoning in cattle (38). Thiamine deficiency affects monogastric animals such as horses and, to a lesser extent, pigs that receive bracken as part of their diet. Thiamine deficiency is caused by bracken fern thiaminases and is characterized by central nervous system lesions and related symptoms (39). Progressive retinal degeneration is typically observed in sheep that graze on bracken fern and was reproduced in laboratory conditions by administering bracken or ptaquiloside (40, 41). Bovine enzootic hematuria and upper alimentary tract carcinomas are of particular interest for the purposes of this review, because their pathogenesis involves complex interactions between bracken fern toxins and BPV, revealing their partnership as chemical and biological carcinogens. These two particular syndromes will be dealt with in the following sections.

## Bovine Papillomavirus

BPV infections have been observed in cattle in multiple locations worldwide, often with significant effects on livestock production, as well as on the local economy, as previously reviewed by our group and others (2, 42). Among the main genes of PVs, the E2 gene has important function in neoplasms associated with PV in both animals and humans, because the interruption of E2 results in the uncontrolled expression of E6 and E7, which promotes cell transformation (2). The presence of the E1, E2, E3, E4, E5, E6, E7, and E8 genes varies depending on genera. The E1 protein is considered the primary viral replication protein, while E2 acts as a transactivator or enhancer (43). The E3 protein has no known function yet (44). The E4 protein is responsible for viral maturation and is associated with the disruption of the cytoskeleton structure (45). The E5 protein is an important BPV transforming protein and promotes the proliferation of infected cells via activation of the platelet-derived growth factor receptor beta (PDGFRβ) (46) while also promoting immune evasion by down-regulating the expression of major histocompatibility complex type I proteins in the cell surface (47). BPV E5 also interferes with cellular homeostasis by hampering the functions of cytosolic organelles, e.g., by interfering with the acidification of the Golgi complex (48). The E5 protein also forms tri-component complexes with PDGFRβ and the V_1_-ATPase D subunit, which are hypothesized to interfere with cellular proteostasis and autophagy (49). Furthermore, the BPV E5 protein constitutively activates c-Src (50) and activates phosphatidylinositol-3-kinase (PI3K) signaling independently of PDGFRβ (51). The E6 protein promotes the disruption of the actin cytoskeleton increasing cell motility, induces DNA breaks and neosis, p53 downregulation allowing uncontrolled proliferation in the presence of genomic instability, and contributed for metabolic deregulation, resulting in oxidative stress (52). The E7 protein promotes cell transformation by downregulating the retinoblastoma protein (pRb), deregulating the cell cycle and inducing mitosis (53). The E8 gene encodes a small hydrophobic polypeptide that contributes to cell transformation by providing anchorage-independent growth (54). The first PV genotype identified in domestic cattle (*Bos taurus*) (BPV-1) was described in the scientific literature in 1982 (55). Since then, research on this field brought to light other BPV genotypes and their number currently stands at 29. These genotypes are divided into five different genera: *Delta* (δ), *Dyokappa, Yoxi, Epsilon* (ε) and *Xi* (ξ) (56) as detailed in Table 1. There are 15 BPV genotypes classified as *Xipapillomavirus* that have cutaneous association (6, 57). BPV-5 and BPV-8 are classified as *Epsilonpapillomavirus* and are also detected in cutaneous papillomas and fibropapilomas (58). Skin papillomas induced by BPV are histologically benign and generally undergo spontaneous regression. BPV classified as *Deltapapillomavirus* have a unique characteristic which is their ability to cross-infect other species, including different species of animals within the Bovidae, Cervidae, Equidae, Felidae and Girafiidae families (59–66). Viruses of the genus *Deltapapillomavirus* are capable of causing sarcoids in distantly related hosts, such as horses, mules, African lions (67), domestic cats (65), mountain zebras, giraffes and black antelopes (68, 69). These sarcoids are locally invasive spindle-cell skin tumors, histologically characterized by epidermal hyperplasia and subepidermal proliferation of cells that are similar to fibroblasts (66). BPV-16, BPV-18 and BPV-22 are classified as *Dyokappapapillomavirus* and are detected in cutaneous papillomas and vulvovaginitis (57, 70, 71). BPV-7 also belongs to *Dyokappapapillomavirus* and has been identified in cutaneous papilloma and healthy skin. BPV-19, BPV-21 and BPV-27 currently remain unclassified (6). BPV-4 is associated with papillomas and squamous cell carcinomas in the upper digestive tract, including the oral and pharyngeal cavities, the esophagus and the rumen (72). BPV-1 and BPV-2 are associated with epithelial and mesenchymal urinary bladder tumors (Figure 1B) in cattle (73–75) and buffaloes (73).

**Table 1:** BPV types and their associated lesions at multiple anatomic sites.

**BPV type**	**Genus**	**Tissue tropism**	**Anatomical distribution**	**Lesions**	**Early genes**	**References**
BPV 1	*Deltapapillomavirus*	Epithelium and Dermis	Skin, Bladder, Udder	Fibropapillomas Urinary bladder cancer	E1, E2, E4, E5, E6, E7,	(55)
BPV 2	*Deltapapillomavirus*	Epithelium and Dermis	Skin, Bladder, Udder	Fibropapillomas Urinary bladder cancer	E1, E2, E3, E4, E5, E6, E7, E8,	(76)
BPV 3	*Xipapillomavirus*	Epitheliotropic	Skin, Teat, Udder	Epithelial papillomas	E1, E2, E4, E7, E8,	(42)
BPV 4	*Xipapillomavirus*	Epitheliotropic	Skin, Upper digestive tract	Epithelial papillomas Upper digestive carcinomas	E1, E2, E3, E4, E7, E8	(77)
BPV 5	*Epsilonpapillomavirus*	Epithelium and Dermis	Teat	Epithelial papillomas and fibropapillomas	E1, E2, E6, E7	(78, 79)
BPV 6	*Xipapillomavirus*	Epitheliotropic	Skin, Teat	Epithelial papillomas	E1, E2, E4, E7, E8,	(80)
BPV 7	*Dyoxypapillomavirus*	Epitheliotropic	Skin, Teat	Epithelial papillomas	E1, E2, E4, E6, E7	(81)
BPV 8	*Epsilonpapillomavirus*	Epithelium and Dermis	Skin, Teat	Epithelial papillomas and fibropapillomas	E1, E2, E4, E5, E6, E7	(58)
BPV 9	*Xipapillomavirus*	Epitheliotropic	Skin, Teat, Udder	Epithelial papillomas	E1, E2, E5, E7	(82)
BPV 10	*Xipapillomavirus*	Epitheliotropic	Skin, Teat, Udder	Epithelial papillomas	E1, E2, E4, E5, E7	(82)
BPV 11	*Xipapillomavirus*	Epitheliotropic	Teat	Epithelial papillomas Fibropapillomas	E1, E2, E4, E7, E10	(83)
BPV 12	*Xipapillomavirus*	Epitheliotropic	Tongue	Epithelial papillomas	E1, E2, E4, E7, E8	(84)
BPV 13	*Deltapapillomavirus*	Epithelium and Dermis	Skin	Fibropapillomas	E1, E2, E4, E5, E6, E7	(85)
BPV 14	*Deltapapillomavirus*	Epithelium and Dermis	Skin, bladder	Fibropapillomas	E1, E2, E5, E6, E7	(65, 86)
BPV 15	*Xipapillomavirus*	Epitheliotropic	Skin	Epithelial papillomas	E1, E2, E5, E7	(87)
BPV 16	*Dyoxypapillomavirus*	Epithelium and Dermis	Skin	Fibropapillomas and cutaneous papillomas	E1, E2, E4, E6, E7	(88)
BPV 17	*Xipapillomavirus*	Epitheliotropic	Skin	Epithelial papillomas	E1, E2, E6, E7	(88)
BPV 18	*Dyoxypapillomavirus*	Epitheliotropic	Skin	Epithelial papillomas	E1, E2, E6, E7	(88)
BPV 19	Unassigned genus	Epitheliotropic	Skin	Epithelial papillomas	E1, E2, E6, E7	(88)
BPV 20	*Xipapillomavirus*	Epitheliotropic	Skin	Epithelial papillomas	E1, E2, E4, E6, E7	(88)
BPV 21	Unassigned genus	Epitheliotropic	Skin	Epithelial papillomas	E1, E2, E6, E7	(88)
BPV 22	*Dyoxypapillomavirus*	Vaginal mucosa	Vagina	Vulvovaginitis	E1, E2, E6, E7	(70)
BPV 23	*Xipapillomavirus*	Epitheliotropic	Skin	Epithelial papillomas	E1, E2, E4, E7, E8	(56)
BPV 24	*Xipapillomavirus*	Epitheliotropic	Skin	Epithelial papillomas	E1, E2, E4, E5, E7	(89)
BPV 25	*Epsilonpapillomavirus*	Epitheliotropic	Skin	Epithelial papillomas	E1, E2, E6, E7	(90, 91)
BPV 26	*Xipapillomavirus*	Not reported	Not reported	Papillomas	E1, E2, E4, E5, E7, E8	(92)
BPV 27	Unclassified	Not reported	Genitalia	Not reported	E1, E2, E6, E7	(93)
BPV 28	*Xipapillomavirus*	Epitheliotropic	Vulva	Papillomas	E1, E2, E4, E7, E10	(94)
BPV 29	*Xipapillomavirus*	Epitheliotropic	Vulva	Papillomas	E1, E2, E4, E7, E10	(94)

## Bracken Fern And Bpv In Upper Digestive Carcinogenesis

Infection with BPV-4 has been associated with papillomas and squamous cell carcinomas of the upper digestive tract in cattle (95–98). Infection with BPV-4 is associated with oral, esophageal and ruminal papillomatosis and there have been reports of a papilloma-to-carcinoma transition (98). However, no BPV4 DNA or viral oncoproteins could be detected in malignant lesions (99, 100), raising questions about the etiological role of BPV-4 in upper digestive cancer. Exposure to bracken fern may promote the papilloma-to-carcinoma transition as previously suggested (46, 72, 98), with bracken fern toxins cooperating with BPV-4 to achieve a malignant phenotype (Figure 1C), but may also cause upper digestive cancer by itself, without BPV-4 involvement (99). Interestingly, recent findings show that ptaquiloside promotes carcinogenesis initiated by human papillomavirus type 16 (HPV-16) in the oral and pharyngeal cavities of HPV-16-transgenic mice (101). Bracken fern illudane glycosides like ptaquiloside have been proposed to contribute for carcinogenesis in two main ways. The first involves their genotoxic effects, as these compounds are able to alkylate DNA and induce point mutations, namely activating mutations at the Harvey rat sarcoma (H-ras) proto-oncogene codons 58, 58 and 61 (102, 103), which lead to increased cell proliferation. Ptaquiloside also induces chromosome aberrations (104, 105) that ultimately lead to a neoplastic phenotype, as previously reviewed (24). The second way involves their immune suppressive properties, which may facilitate the persistence of oncogenic viruses like BPV and HPV and may also allow early neoplastic lesions to evade immunological surveillance and develop into overt cancers (27). Latorre and collaborators showed that ptaquiloside is able to inactivate natural killer (NK) cells (106), which are key players in innate immunity and are essential for defending the host against viral infections and against cancer (107). When activated, NK cells are able to recognize and destroy neoplastic cells and virus-infected cells. So, by inactivating NK cells, ptaquiloside may facilitate the persistence of BPV infections and the development of early BPV-induced lesions into invasive cancers. More recently, our group showed that ptaquiloside is also able to inactivate cytotoxic T lymphocytes, another cell population involved in adaptive immunity against neoplastic and virus-infected cells (108). Interestingly, the immunotoxic effects of ptaquiloside against NK cells in mice could be reversed with selenium administration (109), suggesting that prevention of bracken fern toxicities can be achievable through dietary modifications. Very recently, the E5 oncoprotein of Delta BPVs was shown to impair the production of type I interferon and the innate antiviral response via interactions with the tripartite motif-containing 25 (TRIM25) protein (110), further establishing the immune evasion mechanisms employed by BPV.

## Bracken Fern And Bpv In The Etiology Of Bladder Tumors

Ingestion of bracken fern has been associated with enzootic hematuria of grazing cattle herds since the 1960s (111, 112). Since then, the occurrence of bladder tumors in cattle grazing on bracken fern has been described by different teams in multiple continents (72, 96, 113–116). The morphology of bovine urinary bladder tumors is varied, includes multiple histological types of epithelial, mesenchymal and mixed lesions, and has been previously described in detail (114–116). The experimental administration of bracken fern to cows reproduced the urinary lesions in controlled conditions (117). In a seminal work that largely sparked research in bracken fern-related carcinogenesis, Evans and Mason (19) showed that bladder neoplasia can also be induced in rats by a diet containing bracken (19). These findings were later reproduced by other teams (17, 118, 119) and also by researchers working with guinea pigs (120). Mutations of the Harvey rat sarcoma virus (H-ras) oncogene were found in rats exposed to ptaquiloside (102, 103), but do not seem to be frequent in bovine bladder tumors (114). Interestingly, the E-ras protein is overexpressed in bovine bladder tumors, binds to PDGFRβ and the available experimental data suggest it may contribute for tumorigenesis via Akt signaling (121). Additional studies are warranted to clarify the role of Ras in bovine bladder carcinogenesis. In mice, exposure to ptaquiloside or bracken fern is mainly associated with lymphoid malignancies (122, 123), but bladder urothelial dysplasia was observed upon ptaquiloside administration (123, 124). BPV-1 and BPV-2 (55, 125) have long been associated with urinary bladder tumors (126–129). More recently, BPV-13 (86, 130) and BPV-14 (131) have also been found in bovine urinary bladder tumors. Interestingly, recent data indicate that specific major histocompatibility complex class II alleles may protect against BPV infection and reduce the risk of bladder cancer in cattle (132). The BPV-1 E5 oncoprotein shows transforming properties *in vitro* and is able to induce cytoskeletal remodeling of murine and bovine cells (133). Unlike in human papillomavirus and other BPVs, the E5 oncoprotein is considered the main transforming protein of delta BPV genotypes, and is able to exert important cell functions such as down-regulating major histocompatibility complex proteins promoting immune evasion (47) and of activating the platelet-derived growth factor receptor beta, driving cell proliferation (134), as previously reviewed (12). Although papillomavirus in general show a tropism for stratified squamous keratinizing epithelia of the skin and mucosae, BPVs are believed to establish non-productive infections of the bladder urothelium. Expression of BPV E5 and E6 has been demonstrated in urinary bladder tumors (75, 97, 131), further supporting BPV's etiological role. Besides its established carcinogenic roles discussed in previous sections, the E5 oncoprotein has recently been shown to interact with E-ras and increase mitophagy in bovine bladder cancer (135), The fact that delta BPVs are able to transform stromal cells also lends support to their etiological role in bladder carcinogenesis, which is frequently characterized by mesenchymal or mixed epithelial-mesenchymal tumors. In fact, BPV-1-transgenic mice develop sarcoid-like mesenchymal lesions of the skin (136–138), but there are no reports of bladder lesions in this mouse model. However, BPV DNA may be detected in non-humoral bladder samples and BPV-negative cases of bladder cancer do occur (74), suggesting that bracken fern is a critical etiological factor. These observations call for additional studies on the interplay between BPV and *Pteridium* sp. in bladder carcinogenesis.

## Conclusion

The 29 BPV types currently recognized induce a variety of lesions in different anatomic sites, some of which are associated with significant economic losses. In the case of BPV-4 and upper digestive tumors, the bracken toxins, in particular illudane glycosides, seem to act as tumor promoters, both by causing gene mutations and by suppressing the host's immune response, allowing viral persistence and cancer development. Accumulating data shows how ptaquiloside inactivates key immune cell populations involved in innate and adaptive immunity and synergizes with papillomavirus to promote upper digestive carcinogenesis. The co-occurrence of BPV infection and bracken exposure in cattle with bladder tumors is striking, but the precise mechanisms underlying the cooperation of these two factors remain are less clear. Bracken fern seems able to induce bladder tumors by itself, in the absence of BPV, but BPV is likely to enhance the carcinogenic effects of bracken illudane glycosides. On the one hand, the immune suppressive properties of bracken are likely to contribute for viral persistence. On the other hand, BPV can sustain cell proliferation via PDGFβ in the presence of genetic damage induced by bracken illudanes, allowing the accumulation of gene mutations and contributing for carcinogenesis. However, the precise gene targets of bracken toxins, initially thought to include H-ras, require additional clarification.

## Author Contributions

All authors listed have made a substantial, direct and intellectual contribution to the work, and approved it for publication.

## Funding

This work was supported by the Research Center of the Portuguese Oncology Institute of Porto (project no. PI86-CI-IPOP-66-2017); by European Investment Funds by FEDER/COMPETE/POCI-Operational Competitiveness and Internationalization Program, and National Funds by FCT-Portuguese Foundation for Science and Technology under projects UID/AGR/04033/2020, UIDB/CVT/00772/2020 and by Base Funding-UIDB/00511/2020 of the Laboratory for Process Engineering, Environment, Biotechnology, and Energy LEPABE-funded by national funds through the FCT/MCTES (PIDDAC); Project 2SMART-engineered Smart materials for Smart citizens, with reference NORTE-01-0145-FEDER-000054, supported by Norte Portugal Regional Operational Programme (NORTE 2020), under the PORTUGAL 2020 Partnership Agreement, through the European Regional Development Fund (ERDF). BM-F was supported by FCT, 2020.07675.BD.

## Conflict of Interest

The authors declare that the research was conducted in the absence of any commercial or financial relationships that could be construed as a potential conflict of interest.

## Publisher's Note

All claims expressed in this article are solely those of the authors and do not necessarily represent those of their affiliated organizations, or those of the publisher, the editors and the reviewers. Any product that may be evaluated in this article, or claim that may be made by its manufacturer, is not guaranteed or endorsed by the publisher.
