# Corrigendum: *Pteridium spp*. and Bovine Papillomavirus: Partners in Cancer

**DOI:** 10.3389/fvets.2022.860838

**Published:** 2022-02-16

**Authors:** Beatriz Medeiros-Fonseca, Ana Lúcia Abreu-Silva, Rui Medeiros, Paula A. Oliveira, Rui M. Gil da Costa

**Affiliations:** ^1^Centre for Research and Technology of Agro-Environmental and Biological Sciences (CITAB), Inov4Agro, University of Trás-os-Montes and Alto Douro (UTAD), Vila Real, Portugal; ^2^Veterinary Sciences Department, University of Trás-os-Montes and Alto Douro (UTAD), Vila Real, Portugal; ^3^Veterinary Sciences Department, State University of Maranhão (UEMA), São Luís, Brazil; ^4^Molecular Oncology and Viral Pathology Group, Research Center of IPO Porto (CI-IPOP)/Rede de Investigação em Saúde (RISE)@CI-IPOP (Health Research Network), Portuguese Oncology Institute of Porto (IPO Porto)/Porto Comprehensive Cancer Center (Porto.CCC), Porto, Portugal; ^5^Molecular Oncology and Viral Pathology Group, Faculty of Medicine, University of Porto, Porto, Portugal; ^6^Biomedicine Research Center (CEBIMED), Faculty of Health Sciences, Fernando Pessoa University, Porto, Portugal; ^7^Virology Service, Portuguese Institute of Oncology (IPO-Porto), Porto, Portugal; ^8^LEPABE - Laboratory for Process Engineering, Environment, Biotechnology and Energy, Faculty of Engineering, University of Porto, Porto, Portugal; ^9^Post-graduate Programme in Adult Health (PPGSAD), Department of Morphology, Federal University of Maranhão (UFMA), UFMA University Hospital (HUUFMA), São Luís, Brazil

**Keywords:** papillomaviruses, bovine papillomavirus, cancer, molecular epidemiology ptaquiloside, bladder

In the original article, funder FCT, 2020.07675.BD was not included. The corrected **Funding** statement appears below.

## Funding

This work was supported by the Research Center of the Portuguese Oncology Institute of Porto (project no. PI86-CI-IPOP-66-2017); by European Investment Funds by FEDER/COMPETE/POCI-Operational Competitiveness and Internationalization Program, and National Funds by FCT-Portuguese Foundation for Science and Technology under projects UID/AGR/04033/2020, UIDB/CVT/00772/2020 and by Base Funding-UIDB/00511/2020 of the Laboratory for Process Engineering, Environment, Biotechnology, and Energy LEPABE-funded by national funds through the FCT/MCTES (PIDDAC); Project 2SMART-engineered Smart materials for Smart citizens, with reference NORTE-01-0145-FEDER-000054, supported by Norte Portugal Regional Operational Programme (NORTE 2020), under the PORTUGAL 2020 Partnership Agreement, through the European Regional Development Fund (ERDF). BM-F was supported by FCT, 2020.07675.BD.

The authors apologize for this error and state that this does not change the scientific conclusions of the article in any way. The original article has been updated.

## Publisher's Note

All claims expressed in this article are solely those of the authors and do not necessarily represent those of their affiliated organizations, or those of the publisher, the editors and the reviewers. Any product that may be evaluated in this article, or claim that may be made by its manufacturer, is not guaranteed or endorsed by the publisher.

